# Supplementary material for: Down-Regulation of lncRNA MBNL1-AS1 Promotes Tumor Stem Cell-like Characteristics and Prostate Cancer Progression through miR-221-3p/CDKN1B/C-myc Axis
Source: Cancers (Basel). 2022 Nov 24;14(23):5783. doi: 10.3390/cancers14235783 (PMC9739743; doi:10.3390/cancers14235783)

Supplementary Materials

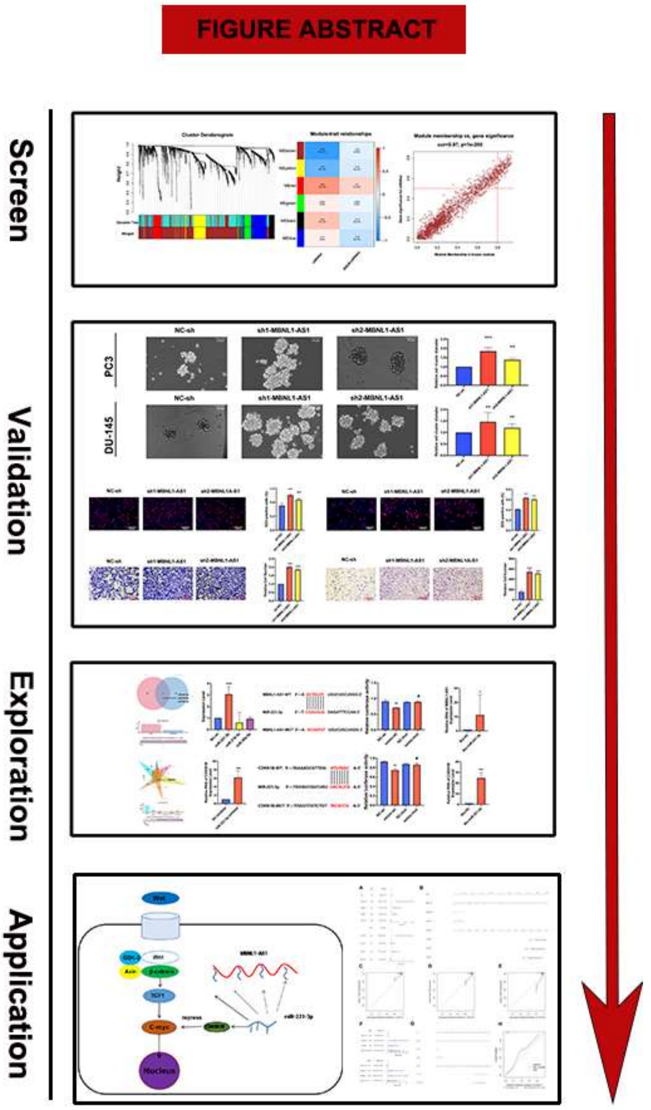

The abstract of figures of this study.

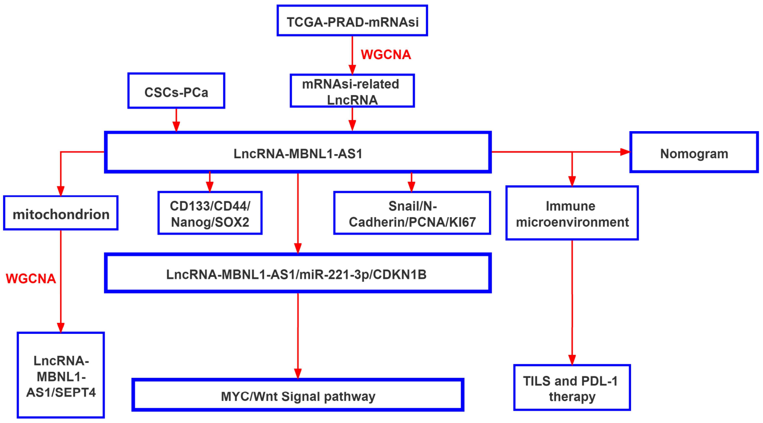

Figure S1. The work flow of this study.

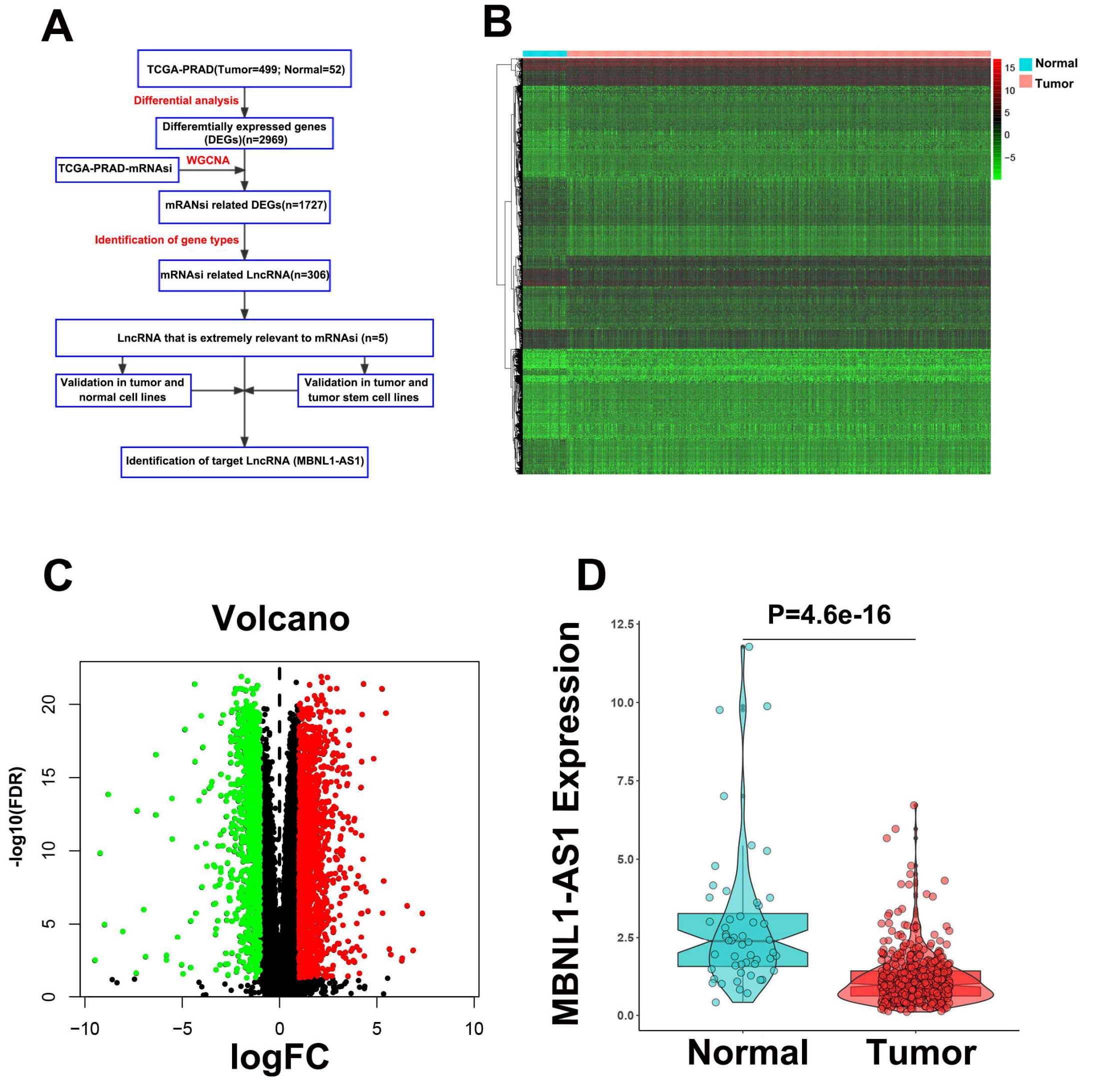

Figure S2. The process of screening. (A) The flow chart of screening the CSC-related LncRNA. (B) Heat map showing expression clustering of differential genes between tumor and normal samples; (C) Volcano plot showing up- vs. down-regulated genes in differential expression analysis, with green dots representing down-regulated genes and red dots representing up-regulated genes; (D) The expression level of MBNL1-AS1 between normal and tumor samples in TCGA-PRAD cohort.

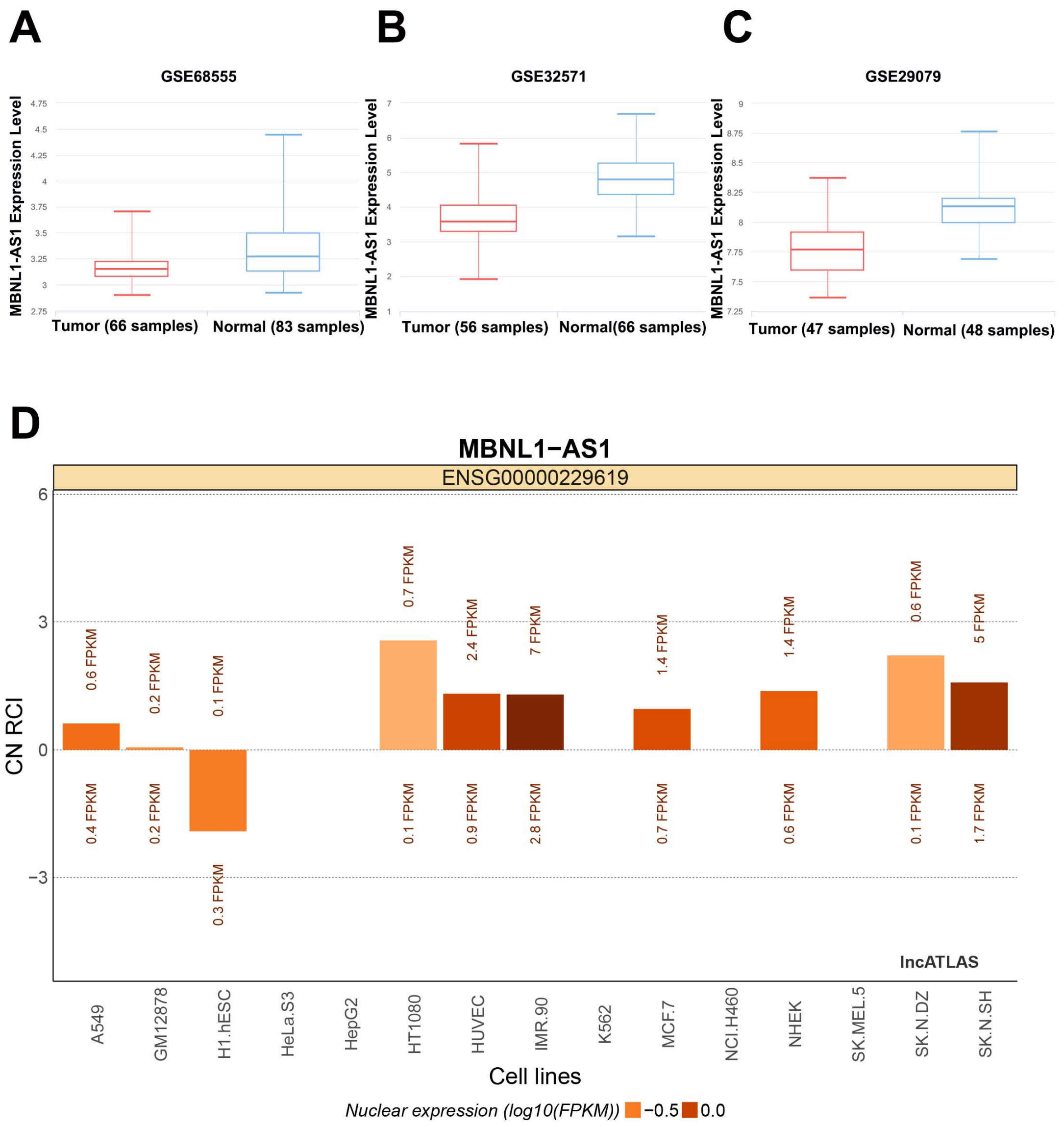

Figure S3. Identification of gene sublocalization and exploration of the effect of target genes on stem cell function. (A-C)The differential profile of LncRNA MBNL1-AS1 between tumor and normal tissues was determined again by combining the GSE68555, GSE32571, and GSE29079 datasets; (D) prediction of LncRNA MBNL1-AS1 by raw signal analysis sublocalization.

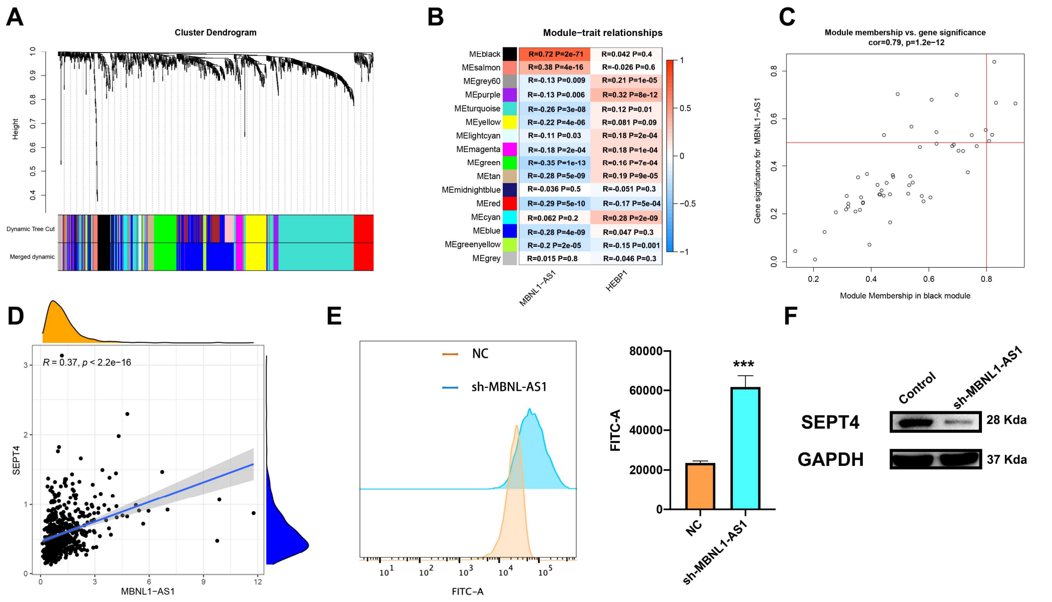

Figure S4. Validation of the correlation between mitochondria-related genes and MBNL1-AS1. (A) Clustering dendrogram of 499 samples. (B) Correlation index of each module with MBNL1-AS1 and HEBP1. (C) Correlation index of genes in the brown module. (D) Correlation analysis of SEPT4 with MBNL1-AS1. (E) The ROS assay was used to detect changes in ROS levels in the cell lines. (F) SEPT4 protein expression levels were assessed by western blot analysis (F). ***P<0.001.

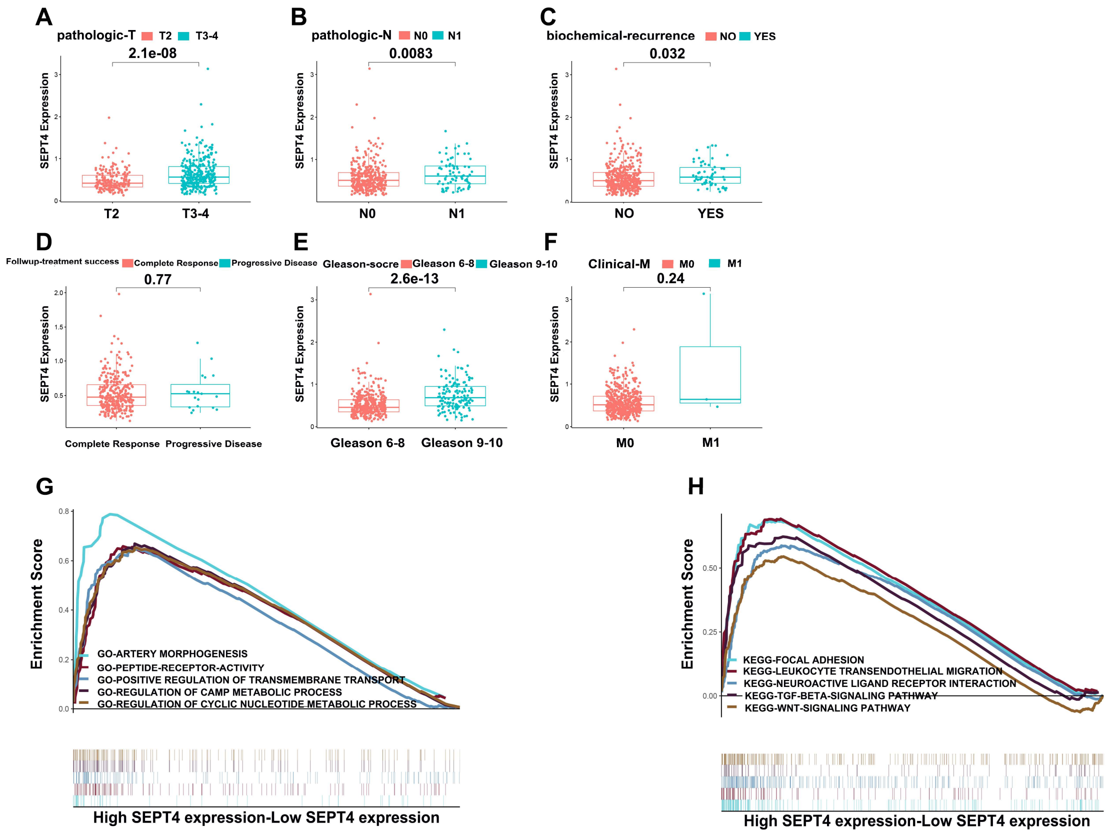

Figure S5. SEPT4 is associated with PCa progression and recurrence. (A-F) Correlation analysis of SEPT4 expression levels with clinical properties. (G-H) Potential functions and pathways associated with SEPT4 were identified by GO and KEGG analyses using GSEA. ‘

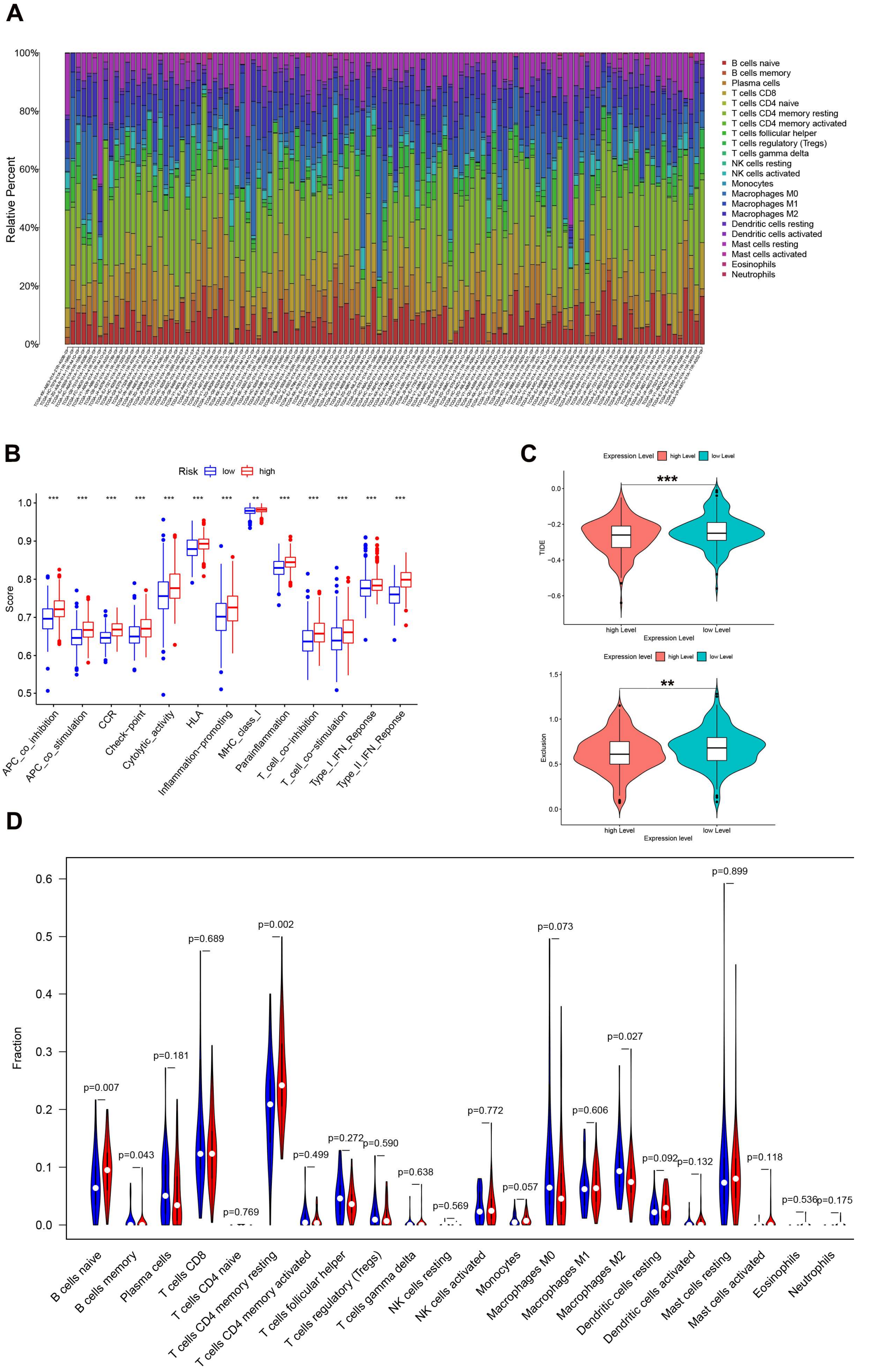

Figure S6. Correlation analysis of LncRNA MBNL1-AS1 and immune system. (A) Bar plot showing the infiltration of immune cells; (B) comparison of the activating of immune response between high-expression-level of MBNL1-AS1 group and high-expression-level of MBNL1-AS1 group; (C) comparison of Immunotherapy escapes with ICIs between high-expression-level of MBNL1-AS1 group and high-expression-level of MBNL1-AS1 group; (D) comparison of immune cell infiltration between high-expression-level of MBNL1-AS1 group and high-expression-level of MBNL1-AS1 group. **P<0.01; ***P<0.001; respectively.

TableS1:The detail of primer plasmid and probe in this study

1、Primer sequences

	Forward (5′-3′)	Reverse (5′-3′)
**CD133**	CCTCTGGTGGGGTATTTCTT	AGGTGCTGTTCATGTTCTCC
**CD44**	CATCCCAGACGAAGACAGTCC	TGATCAGCCATTCTGGAATTTG
**Nanog**	CCTCCTCCCATCCCTCATA	TGATTAGGCTCCAACCATACTC
**Oct4**	AACGACCATCTGCCGCT	CGATACTGGTTCGCTTTCTCT
**MBNL1-AS1**	CTCCCGCTTCTTCTACCGAC	TTGGTGCATTTTAAGGCGGC
**MIR100HG**	CCCAGTGCAAGGACAAAGA	GCAGAGGAGGTGTCTTCAGG
**ADAMTS9-AS2**	CAGAAGGGGCTTGGTTGG	TCGTGTTCCTACCCTATTTTGA
**MAGI2-AS3**	CTACAGCACAGCTGATGAAAG	AGTGTGAAGGTAGGGAGTCTA
**LINC00857**	CGTCTTCCCCTCCATCGTG	GATGGGGTACTTCAGGGTGA
**GAPDH**	GGGAAATTCAACGGCACAGT	AGATGGTGATGGGCTTCCC
**CDKN1B**	AGATGTCAAACGTGCGAGTG	CCATGTCTCTGCAGTGCTTC

2、Plasmid Sequence

Pmirglo-MBNL1-AS1-hsa-miR-221-3p-wt

TCTGGCCTTGTTCCCTTGTTAAACCAGAGGAGTGCATTATTTAAGGAACTTGGCACCATG
GCAGAGCAGAACTGACATCAGTGTCTTCTGACTCTCTCAGGCCATTCATTTTCATTGTCT
TTTTTTTTTTCTTAAAGACAATAAGGACAAGAAGAAGCTCTAAAATAGGCCTAACAGTTTC
CCCAGTTTAATGTAAGGAGGTGCTACATGAGATTTCCAAGAGTATAGTTCTTCCTTCCTC
TGTTTCTTCCTTCTTTCATTTTTTCCTTCTTTTATTCTTTCTATTTTTTCATCCTTTCTTTCTT
CTTTCCATACAGAAAACACTTTTCACACAATGACTCTATTCATTT

Pmirglo-MBNL1-AS1-hsa-miR-221-3p-mut

TCTGGCCTTGTTCCCTTGTTAAACCAGAGGAGTGCATTATTTAAGGAACTTGGCACCATG
GCAGAGCAGAACTGACATCAGTGTCTTCTGACTCTCTCAGGCCATTCATTTTCATTGTCT

TTTTTTTTTTCTTAAAGACAATAAGGACAAGAAGAAGCTCTAAAATAGGCCTAACAGTTTC
CCCAGTTTAATGTAAGGAGGacgatgtaGAGATTTCCAAGAGTATAGTTCTTCCTTCCTCTGT
TTCTTCCTTCTTTCATTTTTTCCTTCTTTTATTCTTTCTATTTTTTCATCCTTTCTTTCTTCTT
TCCATACAGAAAACACTTTTCACACAATGACTCTATTCATTT

Pmirglo- Hsa-miR-221-3p—CDKN1B -wt
ACAGCTCGAATTAAGAATATGTTTCCTTGTTTATCAGATACATCACTGCTTGATGAAGCAA
GGAAGATATACATGAAAATTTTAAAAATACATATCGCTGACTTCATGGAATGGACATCCTG
TATAAGCACTGAAAAACAACAACACAATAACACTAAAATTTTAGGCACTCTTAAATGATCT
GCCTCTAAAAGCGTTGGATGTAGCATTATGCAATTAGGTTTTTCCTTATTTGCTTCATTGT
ACTACCTGTGTATATAGTTTTTACCTTTTATGTAGCACATAAACTTTGGGGAAGGGAGGG
CAGGGTGGGGCTGAGGAACTGACGTGGAGCGGGGTATGAAGAGCTT

Pmirglo- Hsa-miR-221-3p—CDKN1B -mut
ACAGCTCGAATTAAGAATATGTTTCCTTGTTTATCAGATACATCACTGCTTGATGAAGCAA
GGAAGATATACATGAAAATTTTAAAAATACATATCGCTGACTTCATGGAATGGACATCCTG
TATAAGCACTGAAAAACAACAACACAATAACACTAAAATTTTAGGCACTCTTAAATGATCT
GCCTCTAAAAGCGTTGGtacatcgATTATGCAATTAGGTTTTTCCTTATTTGCTTCATTGTACT
ACCTGTGTATATAGTTTTTACCTTTTtacatcgACATAAACTTTGGGGAAGGGAGGGCAGGG
TGGGGCTGAGGAACTGACGTGGAGCGGGGTATGAAGAGCTT

3、FISH probe sequences

	**5′-3′**
**MBNL1-AS1 Homo probe-SA**	GAGCCCTCATTGGATTGCTTCCCACATA

4、Pull down sequences

	**5′-3′**
**hsa-miR-221-3p**	AGCUACAUUGUCUGCUGGGUUUC

pull down probe	

TableS2:The detail of antibody in this experiment

Name	Brand	Number	Working concentration
GAPDH	Abcam	ab8245	1/2000
Acitin	Abcam	ab181557	1/1000
CD133	Abcam	ab222782	1/2000
KLF4	Abcam	ab215036	1/1000
Nanog	Abcam	ab109250	1/2000
OCT4	Abcam	ab181557	1/1000
SOX2	Abcam	ab97959	1/1000
CD44	Abcam	ab254530	1/1000
PCNA	Abcam	ab29	1/1000
KI67	Abcam	ab16667	1/2000
Snail	Abcam	ab216347	1/1000
N-Cadherin	Abcam	ab76011	1/5000
CDKN1B	Abmart	P46527	1/500
C-MYC	Abmart	P01106	1/500
SEPT4	Abmart	O43236	1/500

Figure S7. Original uncropped western blot figures

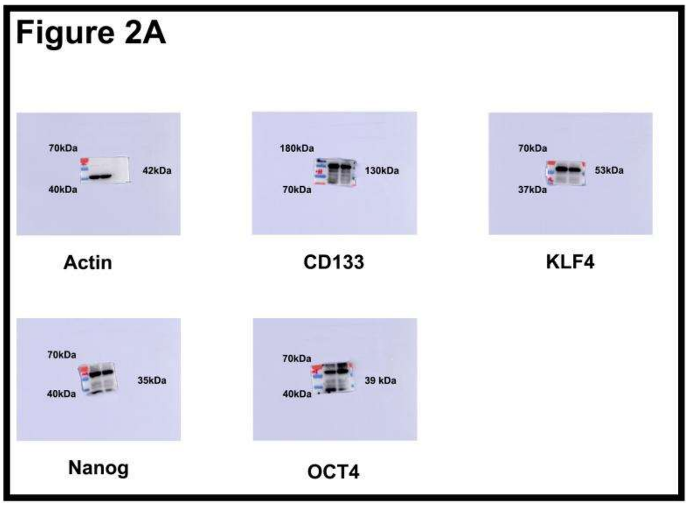

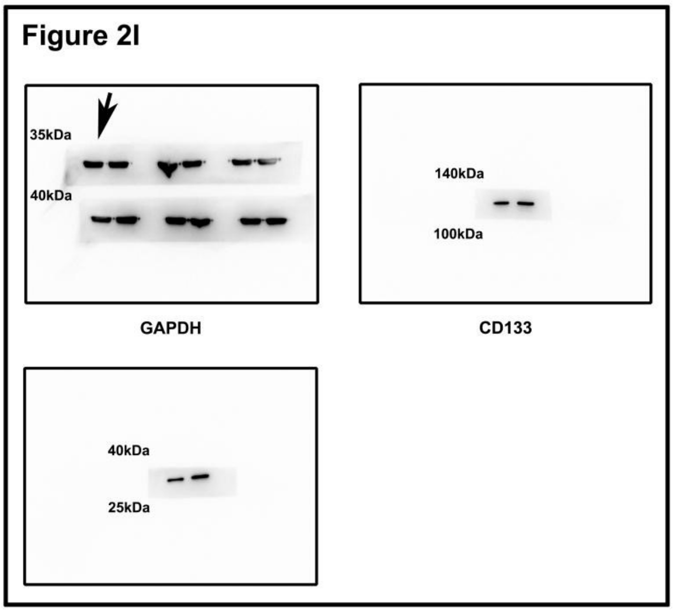

Figure 4N

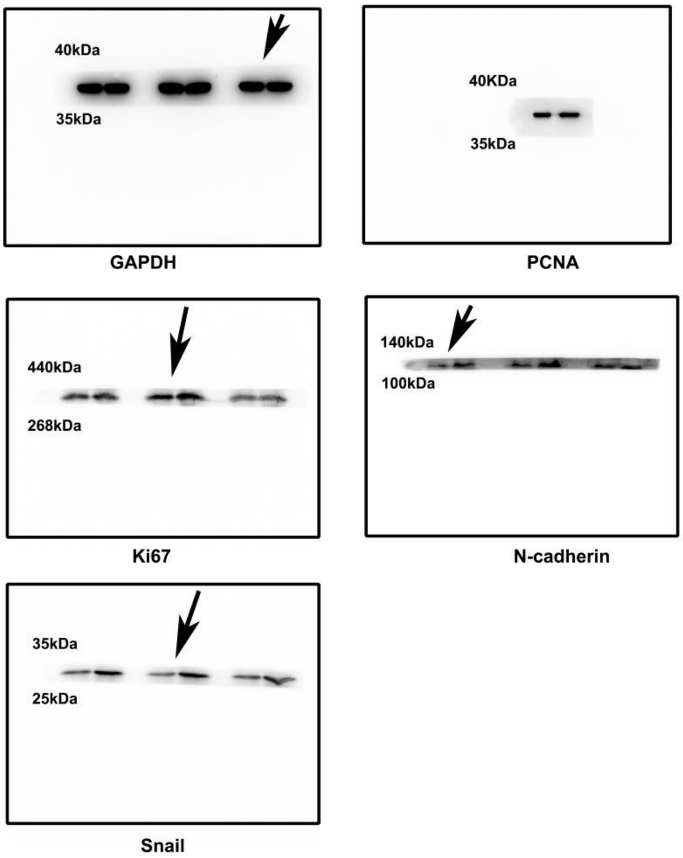

Figure 5N

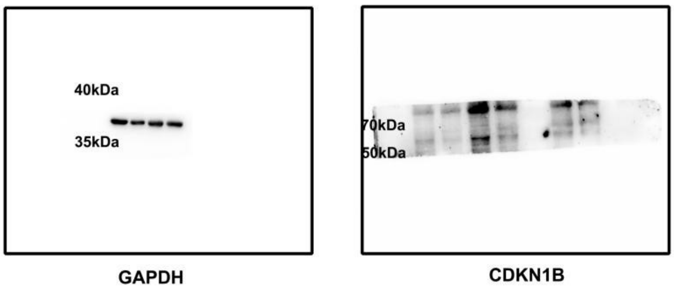

Figure 6I

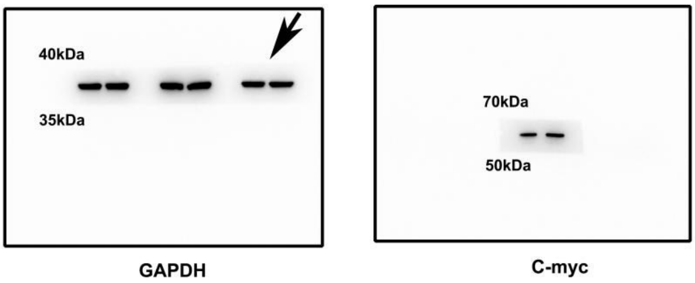

Figure S4F

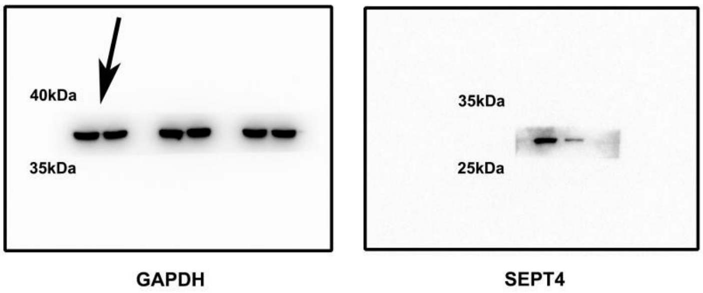

Supplement: Supplementary file 1 [file cancers-14-05783-s001.zip › cancers-2012898-supplementary.pdf]
